# Supplementary material for: The readability of scientific texts is decreasing over time
Source: eLife. 2017 Sep 5;6:e27725. doi: 10.7554/eLife.27725 (PMC5584989; doi:10.7554/eLife.27725)
Supplement: Supplementary file 3. [file elife-27725-supp3.pdf]

Examples of before/after preprocessing of scientific language.

In this document there are 8 examples where we quote the original abstract text, followed by the output from the preprocessing steps which removes many unwanted features of text for quantifying readability. The articles have been selected to demonstrate the effectiveness of the preprocessing steps across disciplines and year of publication.

Example 1 (Annu Rev Immunol, 2008, pmid=17953510):

Original text:

> Interleukin-21 (IL-21), a potent immunomodulatory four-alpha-helical-bundle type I cytokine, is produced by NKT and CD4(+) T cells and has pleiotropic effects on both innate and adaptive immune responses. These actions include positive effects such as enhanced proliferation of lymphoid cells, increased cytotoxicity of CD8(+) T cells and natural killer (NK) cells, and differentiation of B cells into plasma cells. Conversely, IL-21 also has direct inhibitory effects on the antigen-presenting function of dendritic cells and can be proapoptotic for B cells and NK cells. IL-21 is also produced by Th17 cells and is a critical regulator of Th17 development. The regulatory activity of IL-21 is modulated by the differentiation state of its target cells as well as by other cytokines or costimulatory molecules. IL-21 has potent antitumor activity but is also associated with the development of autoimmune disease. IL-21 transcription is dependent on a calcium signal and NFAT sites, and IL-21 requires Stat3 for its signaling. The key to harnessing the power of IL-21 will depend on better understanding its range of biological actions, its mechanism of action, and the molecular basis of regulation of expression of IL-21 and its receptor.

Preprocessed text:

> interleukin a potent immunomodulatory four alpha helical bundle type i cytokine is produced by and cells and has pleiotropic effects on both innate and adaptive immune responses . these actions include positive effects such as enhanced proliferation of lymphoid cells increased cytotoxicity of cells and natural killer cells and differentiation of cells into plasma cells . conversely also has direct inhibitory effects on the antigen presenting function of dendritic cells and can be proapoptotic for cells and cells . is also produced by cells and is a critical regulator of development . the regulatory activity of is modulated by the differentiation state of its target cells as well as by

other cytokines or costimulatory molecules . has potent antitumor activity but is also associated with the development of autoimmune disease . transcription is dependent on a calcium signal and sites and requires for its signaling . the key to harnessing the power of will depend on better understanding its range of biological actions its mechanism of action and the molecular basis of regulation of expression of and its receptor .

Example 2 (Gene Dev, 1997, pmid=9030690):

Original text:

> A prominent feature of cell differentiation is the initiation and maintenance of an irreversible cell cycle arrest with the complex involvement of the retinoblastoma (RB) family (RB, p130, p107). We have isolated the HBP1 transcriptional repressor as a potential target of the RB family in differentiated cells. By homology, HBP1 is a sequence-specific HMG transcription factor, of which LEF-1 is the best-characterized family member. Several features of HBP1 suggest an intriguing role as a transcriptional and cell cycle regulator in differentiated cells. First, inspection of the HBP1 protein sequence revealed two consensus RB interaction motifs (LXCXE and IXCXE). Second, HBP1 interaction was selective for RB and p130, but not p107. HBP1, RB, and p130 levels are all up-regulated with differentiation; in contrast, p107 levels decline. Third, HBP1 can function as a transcriptional repressor of the promoter for N-MYC, which is a critical cell cycle and developmental gene. Fourth, because the activation of the N-MYC promoter in cycling cells required the E2F transcription factor, we show that E2F-1 and HBP1 represent opposite transcriptional signals that can be integrated within the N-MYC promoter. Fifth, the expression of HBP1 lead to efficient cell cycle arrest. The arrest phenotype was manifested in the presence of optimal proliferation signals, suggesting that HBP1 exerted a dominant regulatory role. Taken together, the results suggest that HBP1 may represent a unique transcriptional repressor with a role in initiation and establishment of cell cycle arrest during differentiation.

Preprocessed text:

> a prominent feature of cell differentiation is the initiation and maintenance of an irreversible cell cycle arrest with the complex involvement of the retinoblastoma family . we have isolated the transcriptional repressor as a potential target of the family in differentiated cells . by homology is a sequence specific transcription factor of which is the best characterized family member . several features of suggest an intriguing role as a transcriptional and cell cycle regulator in differ-

entiated cells . first inspection of the protein sequence revealed two consensus interaction motifs and . second interaction was selective for and but not . and levels are all up regulated with differentiation in contrast levels decline . third can function as a transcriptional repressor of the promoter for which is a critical cell cycle and developmental gene . fourth because the activation of the promoter in cycling cells required the transcription factor we show that and represent opposite transcriptional signals that can be integrated within the promoter . fifth the expression of lead to efficient cell cycle arrest . the arrest phenotype was manifested in the presence of optimal proliferation signals suggesting that exerted a dominant regulatory role . taken together the results suggest that may represent a unique transcriptional repressor with a role in initiation and establishment of cell cycle arrest during differentiation .

Example 3 (JAMA, 1982, pmid: 7050440)

Original text:

> The Multiple Risk Factor Intervention Trial was a randomized primary prevention trial to test the effect of a multifactor intervention program on mortality from coronary heart disease (CHD) in 12,866 high-risk men aged 35 to 57 years. Men were randomly assigned either to a special intervention (SI) program consisting of stepped-care treatment for hypertension, counseling for cigarette smoking, and dietary advice for lowering blood cholesterol levels, or to their usual sources of health care in the community (UC). Over an average follow-up period of seven years, risk factor levels declined in both groups, but to a greater degree for the SI men. Mortality from CHD was 17.9 deaths per 1,000 in the SI group and 19.3 per 1,000 in the UC group, a statistically nonsignificant difference of 7.1% (90% confidence interval, -15% to 25). Total mortality rates were 41.2 per 1,000 (SI) and 40.4 per 1,000 (UC). Three possible explanations for these findings are considered: (1) the overall intervention program, under these circumstances, does not affect CHD mortality; (2) the intervention used does affect CHD mortality, but the benefit was not observed in this trial of seven years' average duration, with lower-than-expected mortality and with considerable risk factor change in the UC group; and (3) measures to reduce cigarette smoking and to lower blood cholesterol levels may have reduced CHD mortality within subgroups of the SI cohort, with a possibly unfavorable response to antihypertensive drug therapy in certain but not all hypertensive subjects. This last possibility was considered most likely, needs further investigation, and lends support to some preventive measures while requiring reassessment of others.

Preprocessed text:

> the multiple risk factor intervention trial was a randomized primary prevention trial to test the effect of a multifactor intervention program on mortality from coronary heart disease in high risk men aged to years . men were randomly assigned either to a special intervention program consisting of stepped care treatment for hypertension counseling for cigarette smoking and dietary advice for lowering blood cholesterol levels or to their usual sources of health care in the community . over an average follow up period of seven years risk factor levels declined in both groups but to a greater degree for the men . mortality from was . deaths per in the group and . per in the group a statistically nonsignificant difference of . confidence interval to . total mortality rates were . per and . per . three possible explanations for these findings are considered the overall intervention program under these circumstances does not affect mortality the intervention used does affect mortality but the benefit was not observed in this trial of seven years average duration with lower than expected mortality and with considerable risk factor change in the group and measures to reduce cigarette smoking and to lower blood cholesterol levels may have reduced mortality within subgroups of the cohort with a possibly unfavorable response to antihypertensive drug therapy in certain but not all hypertensive subjects . this last possibility was considered most likely needs further investigation and lends support to some preventive measures while requiring reassessment of others .

Example 4 (Science, 1972, pmid: 5050483)

Original text:

> Horseradish peroxidase (molecular weight, about 40,000) injected into the amniotic sacs in pregnant rats has been identified ultrastructurally, 6 to 18 hours later, within the fetal intestine in the absorptive cells and the underlying vascular endothelium. This indicates that macromolecular protein within amniotic fluid swallowed by the fetus can be absorbed and transported by fetal intestine, and may indicate that physiological compounds can be transported by this enteric route to contribute to fetal development.

Preprocessed text:

> horseradish peroxidase molecular weight about injected into the amniotic sacs in pregnant rats has been identified ultrastructurally to hours later within the fetal intestine in the absorptive cells and the underlying vascular endothelium . this indicates that macromolecular protein within amniotic fluid swallowed by the fetus can be absorbed and transported by fetal intestine and may indicate that physiological

compounds can be transported by this enteric route to contribute to fetal development .

Example 5 (J Clin Invest , 1976, pmid: 175092)

Original text:

> The mean bone pyrophosphate was 0.360 +/- 0.15 mg/g in 8 controls and 1.22 +/- 1.39 mg/g bone in 27 uremic patients (P less than 0.0025). 13 of the 27 uremic patients had bone pyrophosphate levels greater than 2 SD above control values. The ash content of uremic bones with increased pyrophosphate levels (group II) was 56 +/- 9% as compared to 64 +/- 2% in control bones (P less than 0.01) and 60 +/- 7% in uremic bones having normal pyrophosphate levels (P less than 0.1) (group I). The magnesium content of bones in group II was 338 +/- 47 as compared to 211 +/- 13 (P less than 0.0005) in the controls and 294 +/- 73 mmol/kg ash (P less than 0.05) in group I. In group II, but not group I, there was a significant inverse correlation between duration of dialysis and percent bone ash (r = -0.59) (P less than 0.05). A definite relationship existed between elevated bone pyrophosphate levels and soft tissue calcification. In group II the mean pulmonary calcium content was 530 +/- 459 as compared to 32 +/- 26 mmol/kg/ash in group I (P less than 0.0025). All patients with a bone pyrophosphate level greater than 1.4 mg/g bone had extensive pulmonary calcification. It is concluded that the excess bone pyrophosphate present in some uremic patients is either deposited in the apatite crystal in the transphosphorylated form or else as the magnesium salt since the pyrophosphate is resistant to pyrophosphatase and surface adsorption of pyrophosphate is not altered by the increased bone pyrophosphate levels. The excess bone pyrophosphate could disturb bone calcification mechanisms in uremic patients. The association between increased bone pyrophosphate and soft tissue calcification suggests that the disordered pyrophosphate metabolism may be important in the pathogenesis of extraosseous calcification.

Preprocessed text:

> the mean bone pyrophosphate was . in controls and . bone in uremic patients less than . of the uremic patients had bone pyrophosphate levels greater than above control values . the ash content of uremic bones with increased pyrophosphate levels group was as compared to in control bones less than . and in uremic bones having normal pyrophosphate levels less than . group i . the magnesium content of bones in group was as compared to less than . in the controls and mmol ash less than . in group i . in group but not group

i there was a significant inverse correlation between duration of dialysis and percent bone ash . less than . a definite relationship existed between elevated bone pyrophosphate levels and soft tissue calcification . in group the mean pulmonary calcium content was as compared to mmol ash in group i less than . all patients with a bone pyrophosphate level greater than . bone had extensive pulmonary calcification . it is concluded that the excess bone pyrophosphate present in some uremic patients is either deposited in the apatite crystal in the transphosphorylated form or else as the magnesium salt since the pyrophosphate is resistant to pyrophosphatase and surface adsorption of pyrophosphate is not altered by the increased bone pyrophosphate levels . the excess bone pyrophosphate could disturb bone calcification mechanisms in uremic patients . the association between increased bone pyrophosphate and soft tissue calcification suggests that the disordered pyrophosphate metabolism may be important in the pathogenesis of extraosseous calcification .

Example 6 (Trends Ecol Evol, 2014, pmid: 24252439)

Original text:

> The history of life has been characterised by evolutionary transitions in individuality, the grouping together of independently replicating units into new larger wholes: genes to chromosomes, chromosomes in genomes, up to three genomes in cells, and cells in multicellular organisms that form groups and societies. Central to understanding these transitions is to determine what prevents selfish behaviour at lower levels from disrupting the functionality of higher levels. Here, I review work on transposable elements, a common source of disruption at the genome level, in light of the evolutionary transitions framework, and argue that the rapid influx of data on transposons from whole-genome sequencing has created a rich data source to incorporate into the study of evolutionary transitions in individuality.

Preprocessed text:

> the history of life has been characterised by evolutionary transitions in individuality the grouping together of independently replicating units into new larger wholes genes to chromosomes chromosomes in genomes up to three genomes in cells and cells in multicellular organisms that form groups and societies . central to understanding these transitions is to determine what prevents selfish behaviour at lower levels from disrupting the functionality of higher levels . here i review work on transposable elements a common source of disruption at the genome level in light of the evolutionary transitions framework and argue that the rapid influx of data on transposons from whole

> genome sequencing has created a rich data source to incorporate into the study of evolutionary transitions in individuality .

Example 7 (Cell, 2013, pmid: 23582325)

Original text:

> NLRP3 is a key component of the macromolecular signaling complex called the inflammasome that promotes caspase 1-dependent production of IL-1b. The adaptor ASC is necessary for NLRP3-dependent inflammasome function, but it is not known whether ASC is a sufficient partner and whether inflammasome formation occurs in the cytosol or in association with mitochondria is controversial. Here, we show that the mitochondria-associated adaptor molecule, MAVS, is required for optimal NLRP3 inflammasome activity. MAVS mediates recruitment of NLRP3 to mitochondria, promoting production of IL-1b and the pathophysiologic activity of the NLRP3 inflammasome in vivo. Our data support a more complex model of NLRP3 inflammasome activation than previously appreciated, with at least two adapters required for maximal function. Because MAVS is a mitochondria-associated molecule previously considered to be uniquely involved in type 1 interferon production, these findings also reveal unexpected polygamous involvement of PYD/CARD-domain-containing adapters in innate immune signaling events.
>
> is a key component of the macromolecular signaling complex called the inflammasome that promotes caspase dependent production of . the adaptor is necessary for dependent inflammasome function but it is not known whether is a sufficient partner and whether inflammasome formation occurs in the cytosol or in association with mitochondria is controversial . here we show that the mitochondria associated adaptor molecule is required for optimal inflammasome activity . mediates recruitment of to mitochondria promoting production of and the pathophysiologic activity of the inflammasome in vivo . our data support a more complex model of inflammasome activation than previously appreciated with at least two adapters required for maximal function . because is a mitochondria associated molecule previously considered to be uniquely involved in type interferon production these findings also reveal unexpected polygamous involvement of domain containing adapters in innate immune signaling events .

Example 8 (Drugs, 1986, pmid: 3512234)

Original text:

Aztreonam (azthreonam; SQ 26,776) is the first member of a new class of beta-lactam antibiotics, the monobactams. Aztreonam is selectively active against Gram-negative aerobic bacteria and inactive against Gram-positive bacteria. Thus, in vitro, aztreonam is inhibitory at low concentrations (MIC90 less than or equal to 1.6 mg/L) against Enterobacteriaceae except Enterobacter species, and is active against Pseudomonas aeruginosa, 90% of pseudomonads being inhibited by 12 to 32 mg/L. Aztreonam is inactive against Gram-positive aerobic bacteria and anaerobes, including Bacteroides fragilis. Therefore, when administered alone, aztreonam has minimal effect on indigenous faecal anaerobes. Aztreonam must be administered intravenously or intramuscularly when used to treat systemic infections, since absolute bioavailability is very low (about 1%) after oral administration. Since elimination half-life is less than 2 hours, 6- or 8-hourly administration is used in the treatment of moderately severe or severe infections, although 12-hourly injection is adequate in less severe systemic and some urinary tract infections. Therapeutic trials have shown aztreonam to be effective in Gram-negative infections including complicated infections of the urinary tract, in lower respiratory tract infections and in gynaecological and obstetric, intra-abdominal, joint and bone, skin and soft tissue infections, uncomplicated gonorrhoea and septicaemia. In comparisons with other antibiotics, aztreonam has been at least as effective or more effective than cefamandole in urinary tract infections and similar in efficacy to tobramycin or gentamicin. Where necessary, aztreonam and the standard drug have both been combined with another antibiotic active against Gram-positive and/or anaerobic bacteria. Aztreonam has been effective in eradicating pseudomonal infections in most patients (except in patients with cystic fibrosis), but the inevitably limited number of pseudomonal infections available for study prevents any conclusions as to the relative efficacy of aztreonam compared with other appropriate regimens against these infections. Thus, with an antibacterial spectrum which differs from that of other antibiotics, aztreonam should be a useful alternative to aminoglycosides or 'third generation' cephalosporins in patients with proven or suspected serious Gram-negative infections.

Preprocessed text:

aztreonam azthreonam is the first member of a new class of beta lactam antibiotics the monobactams . aztreonam is selectively active against gram negative aerobic bacteria and inactive against gram positive bacteria . thus in vitro aztreonam is inhibitory at low concentrations less than or equal to against enterobacteriaceae except enterobacter species and is active against pseudomonas aeruginosa of pseudomonads being inhibited by to . aztreonam is inactive against gram positive aerobic bacteria and anaerobes including bacteroides

fragilis . therefore when administered alone aztreonam has minimal effect on indigenous faecal anaerobes . aztreonam must be administered intravenously or intramuscularly when used to treat systemic infections since absolute bioavailability is very low about after oral administration . since elimination half life is less than hours or hourly administration is used in the treatment of moderately severe or severe infections although hourly injection is adequate in less severe systemic and some urinary tract infections . therapeutic trials have shown aztreonam to be effective in gram negative infections including complicated infections of the urinary tract in lower respiratory tract infections and in gynaecological and obstetric intra abdominal joint and bone skin and soft tissue infections uncomplicated gonorrhoea and septicaemia . in comparisons with other antibiotics aztreonam has been at least as effective or more effective than cefamandole in urinary tract infections and similar in efficacy to tobramycin or gentamicin . where necessary aztreonam and the standard drug have both been combined with another antibiotic active against gram positive and or anaerobic bacteria . aztreonam has been effective in eradicating pseudomonal infections in most patients except in patients with cystic fibrosis but the inevitably limited number of pseudomonal infections available for study prevents any conclusions as to the relative efficacy of aztreonam compared with other appropriate regimens against these infections . thus with an antibacterial spectrum which differs from that of other antibiotics aztreonam should be a useful alternative to aminoglycosides or third generation cephalosporins in patients with proven or suspected serious gram negative infections .
